# Experiences of Injuries and Injury Reporting among Swedish Skydivers

**DOI:** 10.1155/2014/102645

**Published:** 2014-01-16

**Authors:** Mats Jong, Anton Westman, Britt-Inger Saveman

**Affiliations:** ^1^Department of Nursing, Mid Sweden University, Sundsvall, Sweden; ^2^Mid Sweden Research & Development Centre, Västernorrland County Council, 851 70 Sundsvall, Sweden; ^3^Department of Physiology and Pharmacology, Karolinska Institutet, 171 77 Stockholm, Sweden; ^4^Department of Nursing, Umeå University, 901 87 Umeå, Sweden

## Abstract

The objective was to illuminate the experience of injuries and the process of injury reporting within the Swedish skydiving culture. Data contained narrative interviews that were subsequently analyzed with content analysis. Seventeen respondents (22–44 years) were recruited at three skydiving drop zones in Sweden. In the results injury events related to the full phase of a skydive were described. Risk of injury is individually viewed as an integrated element of the recreational activity counterbalanced by its recreational value. The human factor of inadequate judgment such as miscalculation and distraction dominates the descriptions as causes of injuries. Organization and leadership act as facilitators or constrainers for reporting incidents and injuries. On the basis of this study it is interpreted that safety work and incident reporting in Swedish skydiving may be influenced more by local drop zone culture than the national association regulations. Formal and informal hierarchical structures among skydivers seem to decide how skydiving is practiced, rules are enforced, and injuries are reported. We suggest that initial training and continuing education need to be changed from the current top-down to a bottom-up perspective, where the individual skydiver learns to see the positive implications of safety work and injury reporting.

## 1. Introduction

Skydiving, sport parachuting from aircraft, engages more than 800,000 participants in over one hundred countries who make more than 6 million jumps per year. It is a competitive sport with national and international championships in several disciplines [1].

Skydiving can be viewed as an “extreme sport,” by being associated with a risk of death or serious injury [2]. Skydiving activities are dependent on rules and regulations issued by authorities, but it is also suggested that the policing role of more experienced skydivers sets limits of risk behavior to an even higher degree than formal regulations [3].

The risks associated with modern skydiving have not been satisfactorily described in the scientific literature. Because of lack of unified reporting systems and differences in definitions of injury, the reporting and comparisons of incidents and injuries are hard to make. Injury rates of 170 per 100,000 jumps with an accompanying hospital admission rate of 18 per 100,000 jumps have been reported from the United States [4] and similar rates have been reported from European countries [5–7]. In Sweden, an injury rate of 48 per 100,000 jumps has been reported [8].

Most countries in the world do not have reliable figures on the epidemiology of incidents and injuries. The International Parachuting Commission (IPC) has identified four member countries (France, Finland, Norway, and Sweden) which collect reliable and valid data as the countries have compulsory national routines for reporting of injury events. The IPC has a reporting system for injuries and fatal incidents, but severe problems in acquiring reliable data have made it necessary to identify elements that may influence the reporting culture in the different member states [1]; that is, what makes the individual skydiver choose to abide to formal rules connected to reporting of incidents and injuries.

Studies of Swedish skydiving have used epidemiological designs to describe fatal and nonfatal injury events [8, 9]. A report is compulsory for an incident/injury that leads to a health care visit [10]. The validity of the nonfatal data may be questioned since the performance of the compulsory Swedish system has been reported as having a sensitivity of 0.37 and a specificity of 0.91 [11]. Reasons for this grave underreporting of injuries, several of which were serious or even life threatening, are not known. Social or cultural factors that may play a role are difficult to assess through epidemiological quantitative designs.

A qualitative interview study may provide grounds for understanding and clarifying why individuals, despite the compulsory reporting obligations, choose not to report adverse events [11]. A study from Wales found “culture,” or more specifically, “reporting culture” among surgeons to be one explanatory variable for a discrepancy between surgeons and theatre nurses. Approximately 55% of surgeons and 91.5% of theatre nurses report all or more than 50% of mucocutaneous and percutaneous injuries occurring in operating theatres. The cumbersome administrative procedure, the attitude, personal characteristics of the surgeon, and lack of feedback were given as important reasons for not reporting incidents [12]. According to Roberts and Rousseau [13], can management attitudes and institutional climate greatly influence the success or failure of reporting efforts in high reliability organizations. In high-reliability theory, incident reporting/response systems are important instruments to effectively synthesize and share information with the relevant people across an organization so that appropriate action can be made to prevent or reduce the risk of accidents and disasters [14]. This is also the basic idea for reporting incident and injury events within the sport of skydiving. For instance, a reported incident related to technical failure of a specific piece of the parachute equipment may lead to modification or market withdrawal.

Although most skydivers are pursuing their air sport as a leisure time activity and not as a job, individual, organizational, subcultural (e.g., “reporting culture”), and social aspects may influence the motivation and other incentives to report incidents and injuries. To date, no studies have given voice or studied how skydivers describe events leading to incidents and injury, or how this relates to injury risks and injury reporting.

The objective of the present study was to illuminate the experience of injuries and the process of injury reporting within the Swedish skydiving culture.

## 2. Method

This study is based on a qualitative approach, where data was collected in narrative interviews that were subsequently analyzed with content analysis [15]. A qualitative study can provide a scientific ground to interpret phenomena on the basis of the meanings the studied group of people assign them [16].

### 2.1. Respondents

Inclusion of respondents was made through a convenience sampling procedure; that is, respondents readily at hand and willing to participate in the study were interviewed. Respondents were recruited through a personal contact on three different skydiving drop zones in Sweden. Efforts were made to include respondents from a broad spectrum of skydivers regarding age, sex, and experience of skydiving.

Twenty people were invited to the study; three chose not to participate because of time constraints. The total sample of 17 respondents consisted of six women and 11 men between the ages of 22 and 44 (median = 29). They had been skydiving between one and 25 years (median = 6) and had made between 14 and 3400 jumps (median = 750).

### 2.2. Interviews

Each interview began with a short presentation of the study, followed by a broad question “Could you please tell me about events where you have been injured or have nearly injured yourself while skydiving?” Follow-up questions, if necessary, were asked in accordance with an interview guide (Table 1). Interviews were digitally recorded and lasted between half an hour and two hours and were later transcribed verbatim. The interviews were conducted in places chosen by the respondents, either at drop zones, at respondents' home, in cafés, or at their work.

### 2.3. Data Analysis

The text data underwent a qualitative content analysis where an attempt was made to determine and interpret both the manifest (what is said) and the latent content (what it means) of the text, referred to as categories and/or themes [15, 17]. The description of the analytic procedure is simplified as the three steps that were intertwined and overlapping.

*The First Step*. To achieve an overall understanding of the text, each transcript was read several times. Along with the reading, meaning units were identified and given a temporary code in the body of the text. The coding was used to more easily link and sort text passages connected to each other.

*The Second Step*. During the process of coding and sorting the meaning units, three categories, were identified. In the first category named *descriptions of incidents and injury events,* a coding scheme was used. The scheme that aimed to describe mechanisms in skydiving injuries (phase of jump, mechanism of incident, and mechanism of injury) was developed by Ellitsgaard [6] and further developed by Westman and Björnstig [8]. We used one part of the scheme as a phase of deduction to gain better comprehensive understanding (c.f. Ricoeur [18], page 71–88). The categories *human factor as a contributor to incidents and injuries* and *organization and leadership as facilitators or constrainers for reporting* emerged inductively; that is, no premade coding scheme was used and they were formulated from the manifest content of the text passages relating to incidents and injury events and the reporting of incidents.

*The Third Step*. This step aimed to compare prior steps, codes, and categories with the entire text to obtain a sense of what it is about, to seek and identify a common theme, and to more comprehensively understand and interpret the findings. The identified theme named *skydiving culture—A place for joy, playfulness, and safety awareness* provides a description and an understanding of the cultural context present throughout the prior identified categories.

### 2.4. Ethical and Methodological Considerations

The research performed in this study is not regulated by the Swedish legislation of research on humans [19]. The authors took into account ethical issues that may have appeared through pursuing the study.

Prior to each interview, the interviewer informed the respondent of the study's aim, how long the interview would take, that participation was voluntary, and that the respondent could terminate study participation at any time. The interviewer also informed the respondent that no individual would be named and that respondent integrity and confidentiality would be respected in the presentation of the findings.

In order to enhance trustworthiness and transferability [15], the structuring and analytic procedure was regularly discussed among the three authors. When necessary, codes, categories, and theme were revised, reorganized, added, or omitted to account for the interrelations of the parts and the whole. A second way trustworthiness and transferability [15] was enhanced was through presenting and discussing findings in a group comprised by an expert panel of ten skydiving instructors associated with various skydiving clubs in Sweden. The discussion confirmed the general content of categories and theme and no additional changes were made. In the presentation of quotes, the numbers within parentheses represents the consecutive number of the interviewee; *m* refers to male and *f* to female.

The first two authors (Mats Jong and Anton Westman) are experienced skydivers with approximately 25 years each in the sport. Their preunderstanding was a necessary requisite to conduct interviews with skydivers on their own terms, that is, by sharing the same language and culture. Mats Jong and Anton Westman had no personal connections to the respondents prior to the interview. During the interviews, all respondents were perceived by the interviewers as being able to speak easily and freely even though the subjects related to experiences of incidents, injury, and death. During the analysis, the two interviewers strived to put their preunderstanding in the background to stay open for what came out from the text. The third author (Britt-Inger Saveman) had no previous knowledge or experience in the sport but had extensive knowledge in the methodology used and the topic of injury and injury prevention; thereby, the author has been able to approach the topic without preunderstanding.

In this study, the term “skydiving culture” refers to each respondent's self-defined notion and what it means to them from an individual perspective, including how they experience its relation to injury risks and injury reporting.

## 3. Results

To present an overall understanding of the topic and the context of the skydiving culture, the theme is presented first, followed by the three categories.

### 3.1. Skydiving Culture—A Place for Joy, Playfulness, and Safety Awareness

Skydivers express a constant awareness of imminent injury or death balanced by the positive experiences of being able to pursue the sport. Participating in skydiving activities is interpreted as including a form of risk negotiation where the risk of potential injury is balanced against individual benefits in the form of pleasure, progression, and learning in skydiving. In the narratives, it is possible to identify attitudes of risk behavior differing between males and females; caution is referred to as a feminine characteristic, and the will to develop and progress by pushing limits is referred to as a masculine characteristic. Regardless of the gender of the skydiver, these attitudes toward risk behavior remained the same.

*“It's about the same as anywhere else, girls are not that eager to show off or show how tough they are; girls care more about safety, and at the same time a bit timid and do not dare to test their limits. In that sense, it's a kind of danger too because they need to learn too. In the end, I believe guys turn out as more skillful skydivers, but girls probably think more about how they do things. (1, f, 150 jumps)”*

Skydiving is viewed as a sport where males and females can participate and compete on equal terms, but in relation to risk exposure and behavior a recurrent attitude is that it differs between males and females.

Joy, playfulness, and safety awareness are expressed by both experienced skydivers and those new in the sport. These aspects are associated with several dimensions of interaction relating not only to a sense of belonging to a special group, but also to the direct experience of in-air activities. The aspects of social life and community are also described as important by the respondents:

*“Skydiving contains elements of joy in a higher degree than other sports. Many sports tend to focus on the elite athlete, but even though we have systems of supporting the elite competitors, there is so much that's just playing—just for fun! Many skydivers jump for several years without competing, or even practicing for competition—they do it just for the fun of doing it. I believe that is unusual in other sport activities. (13, f, 300 jumps)”*

Both the degree of playfulness and the degree of safety awareness are interpreted as dependent on the subculture of the local drop zone. Respondents that have skydived abroad express the view that there is a higher degree of safety awareness among Swedish skydivers compared with skydivers in other countries. Safety awareness and playfulness are interwoven in the culture of the sport where fellow skydivers, for example, check each other's equipment prior to boarding the aircraft. A skydiving novice expressed it as:

*“Safety awareness does not make you less cool, it's quite fantastic that people push for safety” (7, f, 90 jumps).*

Skydiving can be dangerous but the experiences of joy and delight outweighs the thoughts of giving up the sport. A view is that “every” skydiver knows that each skydive is a potential injury event or could even lead to death. Risk and injury are considered a constant ingredient in skydiving and the respondents think about it differently. Some want to make the sport as safe as possible, even if it means more regulations are needed while others express “pushing the limits” individually as part of learning what skydiving is or can be. Crossing the limit can lead to injury, but pushing the limit may also lead to learning. An expressed attitude is that the sport and its pursuers are individualists and hence responsible for their own risk-taking acts leading to incidents. Incidents and critical events lead to reflections of one's own and others' risk in the sport. Such incidents can serve as eye openers and affect the attitude toward skydiving. An experienced skydiver puts this into words:

*“Once again, you have an eye-opener and think about the risks in skydiving. I think more of an attitude to life when I think, for example, of an accident like this (a friend's death); you are reminded how fragile life is and how fast it may end. Back again to this respect, an awareness that is so important to hold on to. I am definitely guilty of losing it from time to time, when I just push through, and everything works fine. At the same time, I am so grateful to be here and do what we do, and if that has affected my attitude to skydiving, I'm unsure. Still, it is a reminder to respect what we are doing and not to forget that. (4, m, 2300 jumps)”*

When asked what constitutes “skydiving culture” the respondents often reflect around evening parties and drinking beer. As expressed in the narratives, the safety control system that aimed to prevent incidents with skydivers under the influence of alcohol seems to work properly; often because of self-regulation where individuals stay on the ground when they have drunk too much the previous day. A breath alcohol tester is used to confirm that the skydiver is not intoxicated and ready to jump. Even if the control system works well, the respondents claim that it fails from time to time and some jump despite the control system. Reflecting on skydiving culture, a respondent says:

*“Well I think about case, for example, and it's nice. That's what really constitutes skydiving culture; drinking beer. Unfortunately it is so. I say unfortunately, it is not so serious to tell new students, “ah well, when you're done with your student training, you're supposed to buy beer and case with it.” Some students had a cake instead, really much nicer and more fun. Case is simple, just buy beer, it happens all the time, so it constitutes skydiving culture. (10, m, 800 jumps)”*

Authors note: case is a tradition of buying fellow skydivers “a case of beer” when passing through different check points or levels in the skydiving sport, for example, after completing student training, first 8-way formation, and first jump from hot air balloon.

### 3.2. Descriptions of Incidents and Injury Events

All respondents in this study had experienced incidents or injury incidents associated with skydiving that occurred at different phases of a jump: from aircraft exit, free fall, parachute opening, parachute flight, and landing. During aircraft exit, bruising and minor lacerations are mentioned as well as strained arms when exiting late while the other group members jump off the plane. Several incidents were described as caused by inadequate separation from other skydivers in free fall resulting in or nearly resulting in impact with other skydivers, evoking fear and anger. A respondent describes an incident.

*“Well, there was this scary thing which happened; a kind of close call, some guy opened his parachute below me during a big-way jump (authors' remark; in a formation skydiving jump with 20 other skydivers), which scared me a lot. I was terrified and landed with tears in my eyes. (8, f, 350 jumps)”*

Several incidents happened during parachute opening, for example, when total or partial malfunctions of the main parachute necessitated reserve parachute activation. Examples of malfunctions were twisted lines resulting in violently spinning main parachutes or inability to release steering toggles. In conjunction to malfunctions, injury events have occurred because of reduced plan time for a normal parachute landing, or an increased sink rate because of malfunction or a smaller reserve parachute. Free fall instability during deployment of the parachute has resulted in events described as “horse shoe malfunctions” causing, for example, a sprained ankle while landing under two parachutes.

Incidents associated with wing parachute flight include, for example, downwind landing (high speed—more difficult to come to a stop) resulting in a fractured wrist and several near miss mid-air collision incidents at low altitude. Other examples of incidents include experienced skydivers describing incidents when they miscalculate hook turns, resulting in bruises and fractures. A respondent who fractured an ankle says,

*“Well, once I injured my ankle. I made a turn, kind of low, honestly speaking. I flared too low and hit the grass. It was a hook turn where I waited too long to flare, I landed on my feet but it hurt a lot in one foot. In a way I was lucky, it could have turned out a lot worse if I had been a couple of decimetres lower. (15, m, 1300 jumps)”*

Basically, all respondents gave descriptions of incidents or injury events associated with landing, some resulting in bruises, scratches, sprains, and strains but also more severe injuries including sprained crucial ligaments and fractures of legs and spines. Events occurred both during unintentional and intentional high-speed landings but also in normal low-speed landings. The quote below from a respondent shows an example of the complexity in an injury event while landing outside the drop zone:

*“Well, everything looked just fine. I was planning to land at the nicest spot on the area of clear-cut forest, right in between two tree stumps. It was hard to plan the exact position, but I thought that I could land there. But as I came lower I saw that there was an uprooted tree pointing straight up making the landing area less optimal, at that time I had no choice. I had to land there. My idea was to land in front of one stump and jump over it. It looked just fine, but wasn't under the grass. I stumbled when my feet reached the ground. My feet stuck and my body continued, hands down in full flare (Authors remark; braking), with no chance to catch myself. My head went straight into the tree stump and everything turned black, and I could hear my neck and back crunch. (2, m, 150 jumps)”*

### 3.3. Human Factor as a Contributor to Incidents and Injuries

Inadequate judgment such as miscalculations and failing to pay attention dominates these descriptions as causes to incidents. Additionally, a number of events are described as possibly attributing to female skydivers having less muscular strength than male skydivers.

Miscalculation of exit point from aircraft is described as a factor causing landings outside (forest areas, roads, and in pasture-land) the drop zones (the designated landing area). Another described miscalculation relates to landings at drop zones but where the respondents nearly hit ground obstacles.

A lack of insight into one's own competence and neglecting to undergo proper training about flight characteristics of new equipment was described that as resulting in injuries, for example, using a new wing parachute and realizing that it flew faster at landing than anticipated. Failing in attention and judgment was described as affecting reasons for deviating from set plans, leading to incidents and at times injuries, especially in connection with high speed landings. Parachute traffic, inadequate preparation, and lack of concentration were other reasons mentioned. An experienced respondent gives an example of how different distractions compounded while performing a high speed landing led to an incident:

*“What really happened during that jump was that I deviated from my original plan and began improvising, simultaneously other things happened. I had some kind of attention overload, approaching in a different angle than I was used to, the wind had changed, and additionally there were other parachutes drawing attention. I judged that I would be able to turn around and approach in the direction I was aiming to, which added some extra degrees to my turn, and since I had to pay attention to some other chutes I probably had lost some altitude before I initiated my turn. (4, m, 2300 jumps)”*

A recurrent statement from the respondents is that females and males perform skydiving in different ways. Mentioned by both sexes is the view that females by flying their parachutes too passively do not learn the flying characteristics of their parachute, which may lead to increased risk of injury when encountering situations requiring flexibility and fast unreflective response. An experienced respondent puts this in words by saying,

*“I believe guys hurt themselves for other reason than girls. I can imagine guys hurt themselves more because they are pushing themselves a little bit harder, exceeding their limits. I imagine girls hurt themselves more, not necessarily because they push themselves, but rather because they cannot handle the situation. I believe that if you would expose guys and girls to—it may sound prejudiced, but I believe that if you put guys and girls in exactly the same situations, then girls would suffer more injuries. (6, m, 1400 jumps)”*

Respondents give examples of when they have been unable to deploy their parachute because of not being able to locate the main parachute activation handle or not having the strength to pull it out—resulting in a need to activate the reserve parachute.

### 3.4. Organization and Leadership as Facilitators or Constrainers for Reporting

Whether or not an incident or an injury event is reported to the Swedish Parachuting Association (Svenska Fallskärmsförbundet, SFF) is expressed as depending on a number of distinct reasons. Important emerging issues relate to an individuals' knowledge and definition of what injuries are supposed to be reported, the safety and reporting culture at the local drop zone, organizational structure, and the administration of reports. Additionally, the hierarchical structure in the skydiving culture serves either as a facilitator or a constrainer for reporting. The respondents describe comparable injuries that may be reported in some circumstances but not in others. A recurring view among respondents is that injury severity influences injury reporting propensity. Minor bruises and distortions are described as commonly occurring but rarely reported, whereas major injuries (e.g., fractures) are described as being reported to a higher degree. An important incentive to send in a report is whether the injured person expects long-term consequences, for instance, if the injury may lead to impaired mobility. Respondents report that the regulations are complex and wish for more clarity in some aspects:

*“Today we have all these forms and what is written in the regulations for skydiving, and then it's not, per definition, really an injury (describing a frostbite), more plain stupidity. I consider the regulations good in many ways, but some issues are dense and stupid. And the form for reporting, maybe we could have incidents on three levels: incidents, injuries, and injuries defined as physical injury; that may include frostbite by definition, but I believe it is not done. (3, m, 1300 jumps)”*

An expressed desire is to see the positive implications from reporting and to be given feedback from the skydiving association and the local club on how the administration of reporting benefits the skydiving community. The respondents also express a need to update and modernize the reporting system. Additionally more experienced skydivers need to take more responsibility in teaching newcomers about reporting and safety issues and to a higher degree enforce the formal routines on the drop zones. A novice female respondent expresses it as follows:

*“I think it is laziness which decides if you report or not. You need to fill out all these papers, and that's strenuous. I think you would do it if there's a routine—if I sprain my ankle, someone should come and put that paper in my hand and say that I must do it. If not for that, I would actually not have a thought of reporting. For people like me, you know, I have no routine on how to do it. Other people on the drop zone must show me how to. (7, f, 90 jumps)”*

Although report submission is part of the jump leader's duties, respondents point out that during jump days, the jump leader is quite busy which may constrain reporting of incidents and injuries

Authors note: on a Swedish skydiving drop zone on a given jump day, ultimate responsibility for safety issues is bestowed an assigned instructor, the jump leader. This person is also responsible to make sure that all incidents related to equipment malfunctions, and injuries leading to health care contacts are reported [10].

The role of the jump leader is, according to the respondents, quite unclear among many skydivers, which may contribute to a chaotic situation when incidents occur. Another task of the jump leader, which is highlighted in most interviews, is the commission of restraints as a consequence from unsafe actions or rule violation behavior. What decides if the jump leader acts or not is described as depending on who is reporting and who made the violation. When an experienced skydiver violates regulations resulting in incidents, jump leaders are described as having different incentives not to confront them; sometimes it may be due to an anticipated negative reaction, or they may hesitate because the skydiver is more experienced than themselves. An experienced respondent says,

*“If they would tell me that I am grounded because of unsafe behavior, then I would take the discussion at the end of the day. But, at the same time, if I am aware that what I was doing was wrong, then I would accept the decision. I think it's necessary to enforce the authority and formal assignment, but my opinion is that not many jump leaders do so. The experience level should not be so influential because the rules are clear, you can be experienced or inexperienced and as long as there are rules and regulations you should stick to them. (6, m, 1400 jumps)”*

An expressed view in the narratives is that skydiving clubs in Sweden tend to interpret the national regulations in various ways, which also affects incident reporting. Some clubs are believed to conform to the regulations very strictly and even enforce higher demands than the national regulations on their members by, for example, totally prohibiting high-speed landings. On the other side of the spectrum are clubs, which are considered less conforming to the regulations, informally, allowing people to use equipment not suitable to their experience level.

Depending on the openness of the local drop zone, it is described that skydivers occasionally avoid showing other skydivers that they hurt themselves. Thereby injuries are not reported for fear of exposing themselves as having made a bad judgment or being embarrassed for having poor skills.

## 4. Discussion

The Swedish national rules and regulations regarding safety aspects seem to suggest conformity in the country as a whole regarding skydiving activities, but present results imply the opposite. The respondents express variability between the Swedish clubs (drop zones) regarding safety issues and abidance to national rules and regulations. This is in line with a previous finding of variability between the Swedish clubs regarding reported nonfatal injury rates 1999–2003 [8] and also with findings of Laurendeau and van Brunschot [3], suggesting that the individual skydiver listens more to fellow skydivers than to rules and regulations about safety measures and skill level required to, for example, jump with a specific parachute or start performing high speed landings.

On the individual level, the interpretation is that the risk of injury is viewed as an integrated element of the recreational activity, counterbalanced by its recreational value. The balancing act between risk of injury, death, and recreational value has several implications. In our study respondents claim that they have awareness when their limit is reached and back off when they come too close to the line of danger. However, they simultaneously expressed that challenging the limits is a means of progression and learning. Challenging the limits is described by Lyng [20] as an act of “edgework,” where the person explores the limits of his/her ability and/or the technology used while maintaining enough control to successfully negotiate the edge. Laurendeau and van Brunschot [3] develop this further in the skydiving context, saying that edge workers sustain an illusion of control when they are “crowding the edge.” In high-risk skydiving activities, if injuries do occur, edgework can represent an attempt to push the responsibility away from them, to someone else or some other factor outside themselves. Several experienced respondents in our study who have had incidents and injuries express a high degree of self-responsibility, taking into account that incidents happen to them because of their own poor judgment (human error) or lack of skill. Noticeably, they acknowledge their own deficiencies but might be influenced by interview directions, where they were encouraged to speak from their own self-experienced perspective and not so much from how they perceive the skill of others. The results are similar to Laurendeau and van Brunschot [3], where it also was expressed that skydivers acknowledged occurred incidents as situations for learning and progressing. Our finding is also to some extent contrasting to the study of Laurendeau and van Brunschot [3], where participants expressed a distance between themselves and those who suffered from injury or died, stating that the “other person” was doing things that they would never do. They took a stance of “blaming the victim,” possibly with a subconscious objective to preserve their own sense of control over risks in the sport.

Landing errors due to poorly executed high speed landings have, worldwide, become an important cause of skydiving-related injuries and deaths [1, 21]. Parachute associations and parachute manufacturers' recommend that only highly skilled skydivers perform these maneuvers, but only a limited number of countries or drop zones use regulations to prevent inexperienced skydivers from performing high speed landings [10, 22]. Nevertheless, drop zones, parachute associations, regulatory authorities, and the skydiving community itself have acknowledged the need for skydivers to be better educated about canopy control and are, hence, arranging canopy piloting courses on basic and advanced levels all over the world (http://www.dropzone.com/).

One of the jump leaders' policing duties includes alcohol use, because in Sweden, as in most countries, it is prohibited to skydive under the influence of alcohol and drugs [10]. From our findings, it appears that alcohol consumption and partying are integrated elements in Swedish skydiving culture, as in the North American and Australian skydiving cultures [23–26]. Control systems exist to prevent skydivers from jumping under the influence of alcohol (jump leader, breath testers), but a previous study [9] found that two out of the 37 fatalities in Swedish skydiving 1964–2003 had a blood alcohol level elevated to the point of impairing cognitive functions [27]. Problems and potential risks in skydiving associated with alcohol and illicit drug use need to be more clearly addressed in the safety work within the skydiving community. In line with the discussion above, it may be most important at the local drop zone level.

Worth noting is that respondents have described some incidents relating to the free fall sequence. In Sweden, a hard helmet is compulsory until you have a B-license (>100 jumps after student status) [10], but as a voluntary action most skydivers in Sweden, regardless of rating, use hard helmets to prevent injury [3]. This may be described as a self-policing act. In surfing the reverse is seen, where experienced surfers make the choice not to use head protection, referring to impaired hearing and feeling restrained [28].

From the findings, we can see that inadequate judgment is described as a major cause and contributing factor to incidents. There are other contributors, which conjointly result in an unwanted event. Stressors affecting judgment can emerge during the full sequence of a jump, from prejump activities to landing. When several stressors emerge simultaneously, the person is at risk of making a hasty/bad decision. This is similar to the concept of “sensory overload,” where input of several “never-ending” cognitive stimuli results in mood changes and irrational psychological behavior [29].

The incidents or injury events described in this study chiefly appear to be perceived by the respondents as attributable to human error (i.e., inadequate decision). Reasons for this operator-oriented view among respondents might be connected to their levels of knowledge, insights of the risk associated with the sport, and their innate motivation for pursuing the sport. The complexity of the chain of events leading to an incident can, at times, be connected to the equipment used; for example, the placing of handles for deploying the reserve parachute may be different depending on bodily position. Problems with the technical equipment (airplane, parachute, altimeters, helmets, etc.) are, in general, rare and not explicitly mentioned by the respondents in this study. Technical equipment is controlled for quality and accuracy in order to adhere to industrial standards. Nevertheless, there are equipment-related incidents mentioned by the respondents that possibly occurred due to insufficient muscular strength. Skydivers need to test the deployment systems on the ground from time to time to ensure that they have sufficient muscle strength to activate their parachute, and additionally the parachute industry must continue to monitor their standards to prevent gear-related incidents.

Our results suggest that the skydiving community and subculture have an influence on safety awareness and on reporting of incidents and injuries among Swedish skydivers. It is, in line with high-reliability theory [14], necessary to raise this issue further to understand how subculture can facilitate reporting so that appropriate action can be taken to prevent or reduce the risk of incidents and injuries. Healthcare researchers [12] and other high reliability organizations [13] have similarly put forth issues of subculture and workplace climate factors, such as burdensome administration and lack of feedback, as obstacles for reporting. Barach and Small [30] have, in other types of aviation, noted similar results where fear of reprisal, lack of trust, code of silence, and skepticism are mentioned as reporting barriers.

It may not only be necessary to identify errors for reporting, the people involved in the “culture” need to see the results and how it is used in order to see the meaning of from reporting. In a study by Waring [31], the participating physicians see reporting as pointless, since the “errors are an inevitable and potentially unmanageable feature of medical work.” Another article from the healthcare setting puts forth that incident reporting does have a role in safety work and care processes, by indirectly affecting the attitudes and knowledge of those working there [32]. Barach and Small [30] conclude that confidential incident reporting systems have a decisive role in identifying errors on the system level and that it is necessary to shift from a punitive to a collaborative mindset that seeks to identify and understand the underlying system failures. In that sense, reporting circumvents the culture of blame [31].

In this study, “skydiving culture” as a concept was understood from the participants self-defined understanding. A more comprehensive and complex concept of skydiving culture, including issues of gender, injury risks, risk behavior, and subculture has been scrutinized from a sociological perspective by several authors in the North American and Australian contexts [3, 23, 26, 33], Wade [24], and Brymer [25] in the North American and Australian contexts.

Organization and leadership aspects within the skydiving community can serve both as facilitators as well as constrainers to reporting. Following this line, perhaps a way of addressing safety issues and improving incident and injury reporting would be to work bottom up instead of top down, to initiate/promote discussions at local drop zones, educate jump leaders more thoroughly, and influence all categories of skydivers to reflect on how they pursue skydiving, the related risk taking, and incidents. This has similarities to the conclusions from a review on the safety systems topic within healthcare settings where it was stressed that in order to learn the lesson and take adequate actions inside and across organizations, local and national systems must work together closely giving constant feedback to those involved in reporting incidents [34]. Our proposition to national parachuting associations is to promote a system, preferably web-based, where skydivers can report incidents anonymously and injuries related to all aspects of skydiving, including while aboard aircraft. This is in line with a proposition made by Nilsson et al. [35]. Starting in 2013, the Swedish Parachuting Association (SFF) launched such a system in part because of the contributions from our research group.

The present findings suggest that jump leaders and other instructors act inconsistently when critical situations/incidents occur, for instance, depending on who is reporting and who had a violation. It also appears that respondents (with knowledge of the content) view the regulations as adequate and even express that jump leaders should be more stringent in enforcing them. Power structures might become relevant when the “formal” power of the jump leader is overridden by a skydiver with more experience or higher instructor ratings, thereby holding more “informal power” on the hierarchical ladder. In line with the writings of Laurendeau et al. [3, 23, 26, 33] the hierarchical structures play a substantial role in the Swedish skydiving setting; again they pinpoint variations among the Swedish clubs regarding safety issues and abidance to national rules and regulations.

Because of the qualitative approach and the limited sample size of this study, it is impossible to generalize the findings to the larger population of skydivers on a national or international level in quantitative terms. By presenting quotes as well as validating the analysis in a group of expert skydiving instructors, we have tried to enhance trustworthiness and transferability [15]. Although an effort was made to include a broad range of skydivers with respect to age and gender, the sample has a relatively high level of experience (years of skydiving and number of jumps). This may have affected the data in different ways but probably by enhancing data quality in that experienced respondents have acquired knowledge of the subject matter and have extensive personal experience of incidents and injuries; thereby, they provide reliable accounts of their experiences.

## 5. Conclusion

On the basis of the results presented above, it is interpreted that safety work and incident reporting in Swedish skydiving may be influenced more by local drop zone culture than by the national association policy. Formal and informal hierarchical structures among skydivers seem to dictate how skydiving is practiced, how rules are enforced, and if incidents or injuries are reported. These findings suggest that a change of safety work doctrine may be of value, from a present top-down to a bottom-up perspective, further empowering the individual skydiver. In practical terms, this could mean ensuring a fast, comprehensive bidirectional flow of information between the skydiver and reporting agency after having submitted an incident or injury incident report, thereby giving meaning to both the adverse event and the act of reporting.

## Figures and Tables

**Table 1:** The interview guide.

Yourself as a skydiver	What constitutes skydiving culture for you	Injuries during skydiving?
(i) Favorite way to skydive (ii) Positive and negative aspects as a skydiver (iii) Deliberately putting yourself in potentially dangerous situations (iv) Affected by others to stretch limits	(i) Skydiving culture versus other sport cultures (ii) In your own club, other clubs, internationally (iii) Experiences while skydiving or being on other drop zones	(i) Descriptions of events leading to injury, the injury itself (ii) Emergency procedures (iii) Incidents/near injury events (iv) Thoughts and feelings before and after incidents and injury (v) Injury reported to SFF (vi) If injured, why continue

Risk behavior—yours and others	Differences between male and female skydivers	Safety issues

(i) Reporting, grounding (ii) Discussions on club or national level (iii) Talking to risk takers, what do you say and how do they react	(i) The way to skydive (ii) Risk taking (iii) Attitudes (iv) Injuries (v) Skill and expertise	(i) Strategies for your own safety (ii) Strategies to identify others' safety issues (iii) Suggestions or ideas for improvement of safety issues (iv) Educative efforts (v) Rules and regulations
